# Plastid Phylogenomics and Plastome Evolution of Nandinoideae (Berberidaceae)

**DOI:** 10.3389/fpls.2022.913011

**Published:** 2022-06-30

**Authors:** Shiqiang Song, Dmitriy Zubov, Hans Peter Comes, Haiwen Li, Xuelian Liu, Xin Zhong, Joongku Lee, Zhaoping Yang, Pan Li

**Affiliations:** ^1^College of Life Sciences and Technologies, Tarim University, Alar, China; ^2^Laboratory of Systematic & Evolutionary Botany and Biodiversity, College of Life Sciences, Zhejiang University, Hangzhou, China; ^3^National Academy of Medical Sciences of Ukraine, Kyiv, Ukraine; ^4^Department of Environment & Biodiversity, University of Salzburg, Salzburg, Austria; ^5^College of Life Science, Tonghua Normal University, Tonghua, China; ^6^Shanghai Chenshan Botanical Garden, Shanghai, China; ^7^Department of Environment and Forest Resources, Chungnam National University, Daejeon, South Korea; ^8^Key Laboratory of Biosystem Homeostasis and Protection, Ministry of Education, Zhejiang University, Hangzhou, China

**Keywords:** *Caulophyllum*, *Gymnospermium*, *Leontice*, North America-Eurasia disjunction, phylogenomics, plastome evolution

## Abstract

Subfamily Nandinoideae Heintze (Berberidaceae), comprising four genera and *ca.* 19 species, is disjunctively distributed in eastern North America vs. Eurasia (eastern Asia, Central Asia, Middle East, and southeastern Europe), and represents an ideal taxon to explore plastid phylogenomics and plastome evolution in Berberidaceae. Many species of this subfamily have been listed as national or international rare and endangered plants. In this study, we sequenced and assembled 20 complete plastomes, representing three genera and 13 species of Nandinoideae. Together with six plastomes from GenBank, a total of 26 plastomes, representing all four genera and 16 species of Nandinoideae, were used for comparative genomic and phylogenomic analyses. These plastomes showed significant differences in overall size (156,626–161,406 bp), which is mainly due to the expansion in inverted repeat (IR) regions and/or insertion/deletion (indel) events in intergenic spacer (IGS) regions. A 75-bp deletion in the *ndh*F gene occurred in *Leontice* and *Gymnospermium* when compared with *Nandina* and *Caulophyllum*. We found a severe truncation at the 5’ end of *ycf*1 in three *G. altaicum* plastomes, and a premature termination of *ropC*1 in *G. microrrhynchum*. Our phylogenomic results support the topology of {*Nandina*, [*Caulophyllum*, (*Leontice*, *Gymnospermium*)]}. Within the core genus *Gymnospermium*, we identified *G. microrrhynchum* from northeastern Asia (Clade A) as the earliest diverging species, followed by *G. kiangnanense* from eastern China (Clade B), while the rest species clustered into the two sister clades (C and D). Clade C included three species from West Tianshan (*G. albertii*, *G. darwasicum*, *G. vitellinum*). Clade D consisted of *G. altaicum* from northern Central Asia, plus one species from the Caucasus Mountains (*G. smirnovii*) and three from southeastern Europe (*G. odessanum*, *G. peloponnesiacum*, *G. scipetarum*). Overall, we identified 21 highly variable plastome regions, including two coding genes (*rpl*22, *ycf*1) and 19 intergenic spacer (IGS) regions, all with nucleotide diversity (*Pi*) values > 0.02. These molecular markers should serve as powerful tools (including DNA barcodes) for future phylogenetic, phylogeographic and conservation genetic studies.

## Introduction

Berberidaceae Juss. (Ranunculales), with typical temperate shrubs/perennial herbs and disjunct distributions in the Northern Hemisphere, has attracted the interest of botanists for more than a century (Hutchinson, 1920; Stearn, 1938; Fukuda, 1967; Ying, 1979; Ying et al., 1984; Loconte and Blackwell, 1985; Xiang et al., 2000; Wang et al., 2007; Zhang et al., 2007; Yu and Chung, 2017; Sun et al., 2018; Chen et al., 2020). Within Berberidaceae, three subfamilies, namely Berberidoideae Eaton, Podophylloideae Eaton, and Nandinoideae Heintze, have recently been supported by phylogenomic analyses of whole-plastome sequence data (Sun et al., 2018; Hsieh et al., 2022), hence corroborating earlier phylogenetic inferences based on ‘traditional’ nuclear ribosomal (ITS) and/or plastid DNA markers (*rbc*L: Kim and Jansen, 1996; *ndh*F: Kim et al., 2004; ITS2 + *mat*K, *rbc*L: Wang et al., 2007; ITS + *acc*D, *ndh*F, *rbc*L: Yu and Chung, 2017).

**FIGURE 1:**
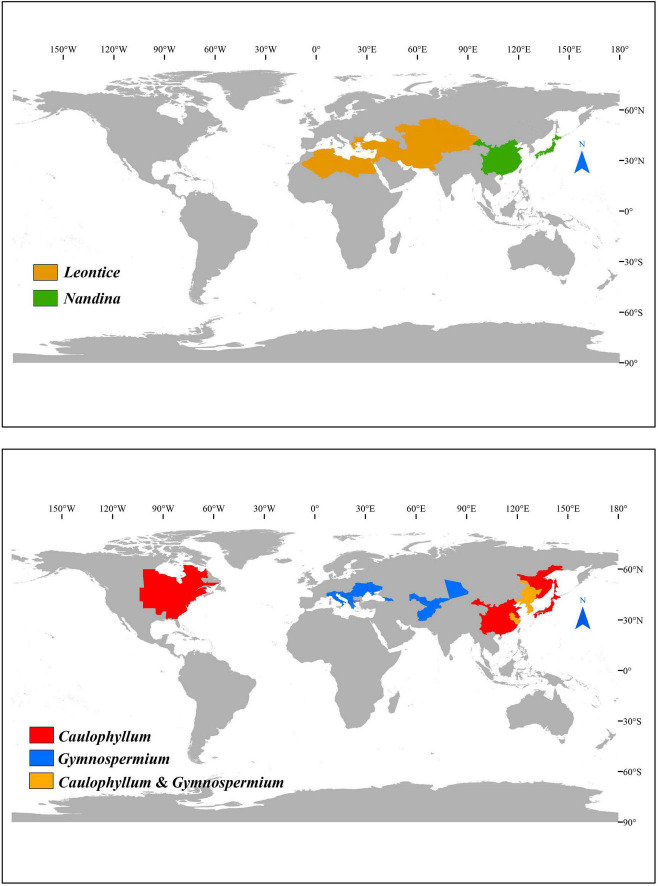
The distribution of Nandinoideae genera.

Here, we focus on subfamily Nandinoideae, which consists of four genera, including *Nandina* Thunb. (1 spp.), *Caulophyllum* Michx. (3 spp.), *Leontice* L. (4 spp.) and *Gymnospermium* Spach (*ca.* 11 spp.). Together, these *ca.* 19 species form a disjunct distribution across the northern temperate zones in eastern North America vs. Eurasia, including eastern Asia, Central Asia, Middle East, and southeastern Europe (Loconte and Estes, 1989; Kim et al., 2004; Wang et al., 2007; Ying et al., 2011; Barina et al., 2017; Rosati et al., 2018; Figure 1). More specifically, the only species of *Nandina*, i.e., *N. domestica* Thunb., is a shrub that occurs in montane forests of China and Japan (Ying et al., 2011; Govaerts et al., 2021). All species of the other three genera are perennial herbs. All three species of *Caulophyllum* grow in mesophytic forests, one (*C. robustum* Maxim.) in eastern China and two [*C. thalictroides* (L.) Michx., *C. giganteum* (Farw.) Loconte and W. H. Blackw.] in eastern North America (Loconte and Estes, 1989; Xiang et al., 2000; Ying et al., 2011). *Leontice* consists of four species, *L. leontopetalum* Hook. fil. and Thomson, *L. armeniaca* Bél., *L. ewersmannii* Bunge and *L. incerta* Pall., all of which are distributed in semiarid to arid regions of Central Asia, Middle East, and southeastern Europe (Loconte and Estes, 1989). *Gymnospermium*, is the most species-rich genus, including about 11 species found in deciduous forests or forest margins, two in eastern Asia [*G. microrrhynchum* (S. Moore) Takht. and *G. kiangnanense* (P. L. Chiu) Loconte], one in the Altai Mountains of Central Asia [*G. altaicum* (Pallas) Spach], four in the west Tianshan Mountains of Central Asia [*G. albertii* (Regel) Takht., *G. darwasicum* (Regel) Takht., *G. vitellinum* M. Král, *G. silvaticum* (Freitag) Takht.] (Barina et al., 2015), one in the Caucasus Mountains [*G. smirnovii* (Trautv.) Takht.], and the remaining three in southeastern Europe [*G. odessanum* (DC.) Takht., *G. peloponnesiacum* (Phitos) Strid, *G. scipetarum* Papar. and Qosja ex E. Mayer and Pulević] (Loconte and Estes, 1989; Ying et al., 2011; Barina et al., 2015, 2017; Rosati et al., 2018; Abidkulova et al., 2021).

Notably, most species of Nandinoideae are rich in alkaloids with important medicinal values (Peng, 2006). For example, in Chinese and Japanese traditional medicine, the roots, stems, leaves, and fruits of *Nandina domestica* are primarily used to treat coughing and asthma (Peng et al., 2014), whereby the fruits are additionally used for the treatment of pharynx tumor, colon cancer and tooth abscess (Hess et al., 1977; Ikuta and Itokawa, 1988; Son et al., 2019). The tubers of *Leontice leontopetalum* and *L. ewersmannii* are used in Turkish traditional medicine for the treatment of epilepsy (Baytop, 1984; Gresser et al., 1993; Kolak et al., 2011; Al-Snafi, 2019). The rhizomes of *Caulophyllum thalictroides* and *C. giganteum* have been used traditionally by Native Americans for inducing childbirth and as anti-inflammatory and anti-pyretic agents (Erichsen-Brown, 1989; Duke, 2002; Rader and Pawar, 2013). Most species of *Gymnospermium* are likewise rich in alkaloids with medicinal properties, such as *G. albertii* (Iskandarov et al., 1967), *G. darwasicum* (Zunnunzhanov et al., 1971), *G. kiangnanense* (Liao et al., 2001), *G. smirnovii* (Tabatadze et al., 2010), and so on. Additionally, many species of *Gymnospermium* have been listed as national or international rare and endangered plants (Chang et al., 2004; Doroftei and Mierla, 2007; Karl and Strid, 2009; Mikatadze-Pantsulaia et al., 2010; Abidkulova et al., 2021).

Plastid phylogenomic analyses have resolved some recalcitrant relationships at various taxonomic levels, for instance among families of early-diverging eudicots (Sun et al., 2016), among genera of Berberidaceae (Sun et al., 2018; Hsieh et al., 2022), and even among species of *Epimedium* Tourn. ex L. (Podophylloideae; Zhang et al., 2016). Comparative plastome analyses have also provided detailed insights into the factors of plastome gene variation and evolution, including gene indel events, expansions and contractions of the inverted repeat (IR) regions, or structural rearrangements (Sun et al., 2016, 2018; Zhang et al., 2016; Ye et al., 2018; Hsieh et al., 2022). Some special structures of plastomes have previously been identified in Berberidoideae and Podophylloideae. For example, 249-bp and 315-bp insertions were seen in the first exon of the *clp*P gene in *Vancouveria hexandra* C. Morren and Decne. and *Epimedium*, respectively (Sun et al., 2016, 2018). Moreover, the plastome of *Bongardia chrysogonum* (L.) Spach was found to contain an inversion of 14 genes (*ca.* 19 kb) in the large single-copy (LSC) region (Sun et al., 2018), while the plastomes of *Berberis* L. and *Mahonia* Nutt. revealed a large expansion in the IR regions (Ma et al., 2013; Sun et al., 2018). Finally, there have been recent reports of a severely truncated *rps*7 gene in the plastomes of several Podophylloideae species, including *Diphylleia sinensis* H. L. Li, *Dysosma versipellis* (Hance) M. Cheng ex T. S. Ying and *Podophyllum peltatum* L. (Sun et al., 2018; Ye et al., 2018).

Within Nandinoideae, only six out of the *ca.* 19 species have fully sequenced plastomes, i.e., *Nandina domestica*, *Caulophyllum robustum*, *Leontice armeniaca*, *L. incerta*, *Gymnospermium microrrhynchum*, and *G. kiangnanense* (Moore et al., 2006; Sun et al., 2018; Yang et al., 2018; He et al., 2019). In this study, we newly sequenced 20 complete plastomes of Nandinoideae, representing three genera and 13 species of this subfamily (including additional accessions of *C. robustum*, G. *microrrhynchum*, and *G. kiangnanense*). Together with the six plastomes previously reported, we included a total of 26 complete plastomes, representing all four genera and 16 species of Nandinoideae (*ca.* 84% of the group’s total species diversity). Based on this extensive plastome dataset, our specific aims were to (1) characterize and compare the structure as well as gene content and order among these plastomes to gain further insights into their evolution; (2) infer phylogenetic relationships within Nandinoideae, especially for the core genus *Gymnospermium*; and (3) identify highly variable plastome regions in Nandinoideae for future phylogenetic, phylogeographic and/or conservation genetic studies.

## Materials and Methods

### Plant Materials and DNA Extraction

For whole plastome sequencing, we collected fresh leaves of 13 Nandinoideae species, including 10 of *Gymnospermium*, one of *Leontice*, and two of *Caulophyllum*, resulting in 20 individuals overall (1–2 individuals per species, see Supplementary Table 1). Voucher specimens were deposited at the herbarium of Tarim University (TARU), Alar, Xinjiang, China (Table 1). Total genomic DNA was extracted from the silica-gel dried leaf tissues using DNA Plantzol Reagent (Invitrogen, Carlsbad, CA, United States), following the manufacturer’s protocol. The quality and quantity of genomic DNA was determined using both 1% agarose gel electrophoresis and an ultraviolet spectrophotometer (K5800, KAIAO, Beijing, China).

**TABLE 1:** Collection locality and voucher information of the Nandinoideae species and accessions used in the present study.

Species and sample code	Collection locality	Voucher information
*C. robustum*	Mengdatianchi National Nature Reserve, Xunhua County, Qinghai Province, China	LP150204-1
*C. thalictroides*	Reeve Rd, Black Earth, Dane County, Wisconsin, US	LP1009205
*L. ewersmannii*	Alai Range, Pamir-Alai, Gulcha vill. vicinities, Osh region, Kyrgyzstan	LP208090
*G. albertii* (I)	M. M. Gryshko National Botanical Garden, Ukraine, originally from Naryn valley, Central Tian Shan, Kyrgyzstan	LP208078-1
*G. albertii* (II)	M. M. Gryshko National Botanical Garden, Ukraine, originally from Naryn valley, Central Tian Shan, Kyrgyzstan	LP208078-2
*G. altaicum* (I)	Yeguolin, Emin County, Xinjiang Province, China	LP173675-1
*G. altaicum* (II)	M. M. Gryshko National Botanical Garden, Ukraine, originally from Zmeinogorsk vicinities, Altai Territory, Russia	LP208079-1
*G. altaicum* (III)	Janis Rukşāns’ nursery, Latvia, Ukraine, originally from Samarskoje vill. vicinities, Kokpekti District, Irtysh river, Kazakhstan	LP208080-1
*G. darwasicum* (I)	Janis Rukşāns’ nursery, Latvia, Ukraine, originally from Vakhsh vicinities, Pamir, Tajikistan	LP208081-1
*G. darwasicum* (II)	Janis Rukşāns’ nursery, Latvia, Ukraine, originally from Karatag vicinities, Gissar Range, Pamir-Alai, Tajikistan	LP208082-1
*G. kiangnanense* (I)	Qimen County, Anhui Province, China	LP184967
*G. microrrhynchum* (I)	Mudanding, Kuandian Manchu Autonomous County, Liaoning Province, China	LP185428
*G. odessanum* (I)	M. M. Gryshko National Botanical Garden, Ukraine, originally from Tiligul estuary, Odessa region, Black Sea Lowland, Ukraine	LP208083-1
*G. odessanum* (II)	M. M. Gryshko National Botanical Garden, Ukraine, originally from Manzyr (Lesnoye) village vicinities, Odessa region, Bessarabian Upland, Ukraine	LP208084-1
*G. peloponnesiacum* (I)	Gothenburg Botanical Garden, Sweden, originally from Achaias, Egialias, Mt. Klokos, NE side ascent from the village of Pteri, Greece	LP208085-1
*G. peloponnesiacum* (II)	Gothenburg Botanical Garden, Sweden, originally from Achaias, Egialias, Mt. Klokos, NE side ascent from the village of Pteri, Greece	LP208085-2
*G. scipetarum* (I)	Gothenburg Botanical Garden, Sweden, originally from Elbasan Mts., 19 km north of Elbasan-Librazhd road, Albania	LP208086-1
*G. scipetarum* (II)	Gothenburg Botanical Garden, Sweden, originally from Elbasan Mts., 19 km north of Elbasan-Librazhd road, Albania	LP208086-2
*G. smirnovii*	M. M.Gryshko National Botanical Garden, Ukraine, originally from Lagodekhi Nature Reserve, Eastern Caucasus, Georgia	LP208087-2
*G. vitellinum*	Janis Rukşāns’ nursery, Latvia, originally from Varzob vicinities, Gissar Range, Pamir-Alai, Tajikistan	LP208088

*Voucher specimens are deposited at the herbarium of Tarim University (TARU), Alar, Xinjiang, China.*

### Genome Sequencing, Assembly, and Annotation

Short-insert (500-bp) paired-end libraries were constructed using the Genomic DNA Sample Prep Kit (Illumina, San Diego, CA, United States), following the manufacturer’s instructions. We used tags to index DNA from each species and pooled samples together for sequencing on a HiSeq™ 2500 platform at the Beijing Genomics Institute (BGI, Shenzhen, China). We assembled raw reads into contigs using NOVOPlasty 2.6.3 (Dierckxsens et al., 2017) with the *mat*K gene of *G. kiangnanense* (MH298010) used as a seed (Yang et al., 2018). The contigs of each Nandinoideae individuals were re-mapped with the reference sequence (MH298010) for whole-plastome assemblage in Geneious Prime^®^ 2021.2.2^1^. For plastome annotation, we used the ‘2544-plastome’ dataset of CPGAVAS2 (Shi et al., 2019) with default parameter settings. All 20 newly sequenced plastomes were illustrated with the online tool OrganellarGenome DRAW v1.3.1 (Greiner et al., 2019) and deposited in GenBank (Table 1). To complement our taxon-sampling, we downloaded six additional plastome sequences of Nandinoideae from NCBI, i.e., *N. domestica* (DQ923117); *C. robustum* (MH423066), *L. armeniaca* (MH423070), *L. incerta* (MH940295), *G. microrrhynchum* (KM057373), and *G. kiangnanense* (MH298010) and reannotated them with above method. Altogether, a total of 16 species and 26 accessions (account for around 84% of Nandinoideae species) were used next comparative plastome analyses.

### Comparative Plastome Analyses

Gene differences among all above 26 Nandinoideae plastomes, including those downloaded from GenBank (see “Genome Sequencing, Assembly, and Annotation”), were performed in Shuffle-LAGAN mode on mVISTA (Frazer et al., 2004). Rearrangements of plastomes were checked by Mauve 2.3.0 (Darling et al., 2004). The structure and junctions between the SSC/LSC regions and the IR regions (see Results) were investigated using IRscope (Amiryousefi et al., 2018). Protein-coding (CDS), intergenic spacer (IGS), and intron regions with alignment length > 200 bp and containing at least one mutation were extracted sequentially. Nucleotide diversity (*Pi*) values for each of those regions were calculated in DNASP v6 (Rozas et al., 2017).

### Phylogenetic Analyses

All 80 protein-coding (CDS) regions of the 26 Nandinoideae plastomes and two outgroups [*Berberis amurensis* Rupr. (KM057374) and *B. weiningensis* T. S. Ying (MW018363)] were extracted and aligned using PhyloSuite v1.2.2 (Zhang et al., 2018) and MAFFT v7 (Katoh and Standley, 2013), respectively. These 80 CDS-genes were then concatenated by PhyloSuite with a set of default parameters. Based on the 80 CDSs, phylogenetic trees of the 28 plastomes (including outgroups) were constructed using maximum likelihood (ML) and Bayesian inference (BI) methods in RAxML-HPC2 on XSEDE v8.2.12 (Stamatakis, 2014) and MrBayes on XSEDE v3.2.7 (Ronquist et al., 2012), respectively. Both analyses are implemented on the CIPRES Science Gateway website^2^. The best-fitting nucleotide substitution model (GTR + I + G) is based on the Akaike Information Criterion (AIC) in jModelTest v2.1.6 (Darriba et al., 2012). For the ML analysis, we set 1,000 bootstrap replicates and defaulted the other parameters. For the BI analysis, we run two independent Markov chain MonteCarlo (MCMC) chains, each for 1,000,000 generations, and sampling every 1,000 generations; the first 25% of the trees were discarded.

## Results

### Plastome Features

**FIGURE 2:**
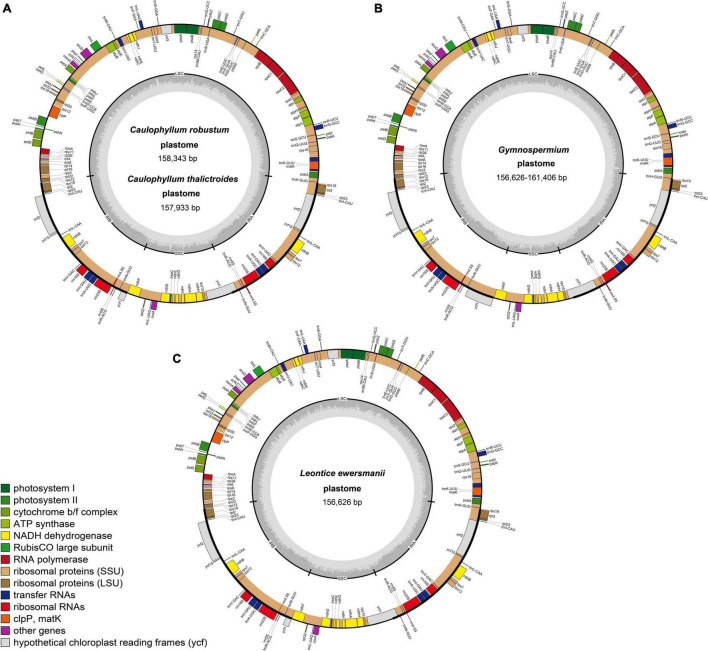
Representative plastome maps of the newly sequenced plastomes of Nandinoideae (20 in total): **(A)** *Caulophyllum robustum and C. thalictroides*, **(B)** *Gymnospermium*, **(C)** *Leontice ewersmannii*.

All 26 plastomes of Nandinoideae, representing 16 species and four genera, exhibited the typical angiosperm quadripartite structure, including a pair of IR regions (IRa, IRb), separated by SSC and LSC regions (Figure 2). All the 26 plastomes contained 114 unique genes (20 in the IR regions), including 80 protein-coding (CDS) genes, 30 tRNA genes, and four rRNA genes (Supplementary Table 1). Of those 114 genes, 17 contained one intron, while three CDS genes (*clp*P, *rps*12, *ycf*3) possessed two introns. Notably, we found two separate *rps*12 gene sequences, one located in the LSC and the other in IRa. For 20 newly sequenced plastomes, the lengths varied significantly, ranging from 156,626 bp in *Leontice ewersmannii* to 161,406 bp in *Gymnospermium smirnovii* (I) (Table 2). The average coverages of the 20 plastomes ranged from 179 × (*Caulophyllum thalictroides*) to 17,448 × [(*G. scipetarum* (I)] (Table 2). The LSC ranged from 85,149 bp in *C. thalictroides* to 87,702 bp in *G. microrrhynchum* (KM057373), the SSC from 14,981 bp in *G. smirnovii* (I) to 20,042 bp in *G. microrrhynchum* (I), and the IRs from 26,292 bp in *Caulophyllum thalictroides* to 30,169 bp in *G. smirnovii* (I) (Table 2).

**TABLE 2:** Basic characteristics of the 26 plastomes of Nandinoideae analyzed in this study.

Species	Accession NO.	Av. cov.	Length (bp)				GC content (%)				Gene number			
			Total	LSC	SSC	IR	Total	LSC	SSC	IR	Total	PCG	rRNA	tRNA
*N. domestica*	DQ923117	–	156,599	85,473	19,002	26,062	38.3	36.6	32.6	43.2	134	87	8	37
***C. robustum***	OM912477	398×	158,343	86,160	19,427	26,378	38.1	36.4	31.9	43.1	134	87	8	37
*C. robustum*	MH423066	–	157,347	85,149	194,58	26,370	38.2	36.4	31.9	43.1	134	87	8	37
***C. thalictroides***	OM912478	179×	157,933	85,598	19,683	26,292	38.2	36.6	31.7	43.2	134	87	8	37
*L. armeniaca*	MH423070	–	157,381	86,039	18,654	26,344	38.3	36.6	32.3	43.1	134	87	8	37
***L. ewersmannii***	OM912479	2822×	156,626	85,508	18,490	26,314	38.3	36.6	32.6	43.2	134	87	8	37
*L. incerta*	MH940295	–	156,923	85,622	18,503	26,399	38.3	36.5	32.4	43.1	134	87	8	37
***G. albertii* (I)**	OM912480	14844×	157,959	85,954	19,076	26,467	38.0	36.4	31.6	43.1	134	87	8	37
***G. albertii* (II)**	OM912481	12784×	157,958	85,953	19,071	26,467	38.0	36.4	31.6	43.1	134	87	8	37
***G. altaicum* (I)**	OM912496	2224×	158,171	86,301	19,092	26,389	38.1	36.3	31.8	43.2	134	87	8	37
***G. altaicum* (II)**	OM912482	10456×	158,076	86,099	19,111	26,433	38.0	36.4	31.6	43.1	134	87	8	37
***G. altaicum* (III)**	OM912483	11425×	158,345	86,475	19,092	26,389	38.1	36.3	31.8	43.2	134	87	8	37
***G. darwasicum* (I)**	OM912484	15212×	158,584	86,479	19,233	26,436	38.0	36.3	31.4	43.1	134	87	8	37
***G. darwasicum* (II)**	OM912485	15669×	158,529	86,564	19,231	26,367	38.0	36.3	31.4	43.1	134	87	8	37
***G. kiangnanense* (I)**	OM912486	932×	157,954	85,750	19,246	26,479	38.2	36.5	31.6	43.1	134	87	8	37
*G. kiangnanense*	MH298010	–	160,134	87,579	19,679	26,439	37.8	36.1	31	43.1	134	87	8	37
***G. microrrhynchum* (I)**	OM912487	1458×	160,561	87,541	20,042	26,489	37.7	36.1	30.7	43.1	134	87	8	37
*G. microrrhynchum*	KM057373	–	160,533	87,702	19,991	26,420	37.7	36	30.7	43.1	134	87	8	37
***G. odessanum* (I)**	OM912488	11622×	158,234	86,056	19,068	26,555	38.0	36.2	31.7	43.1	134	87	8	37
***G. odessanum* (II)**	OM912489	8340×	158,222	86,064	19,048	26,555	38.0	36.2	31.8	43.1	134	87	8	37
***G. peloponnesiacum* (I)**	OM912490	7853×	158,253	86,204	18,951	26,549	38.0	36.2	31.8	43.1	134	87	8	37
***G. peloponnesiacum* (II)**	OM912491	5060×	158,253	86,204	18,951	26,549	38.0	36.2	31.8	43.1	134	87	8	37
***G. scipetarum* (I)**	OM912492	17488×	157,818	86,147	18,755	26,458	38.1	36.4	31.6	43.1	134	87	8	37
***G. scipetarum* (II)**	OM912493	7240×	157,818	86,147	18,755	26,458	38.1	36.4	31.6	43.1	134	87	8	37
***G. smirnovii***	OM912494	9708×	161,406	86,087	14,981	30,169	37.9	36.4	32.1	41.5	134	87	8	37
***G. vitellinum***	OM912495	5700×	158,563	86,601	19,228	26,367	38.0	36.3	31.5	43.1	134	87	8	37

*Accessions in bold represent newly sequenced genomes (20 in total).*

**FIGURE 3:**
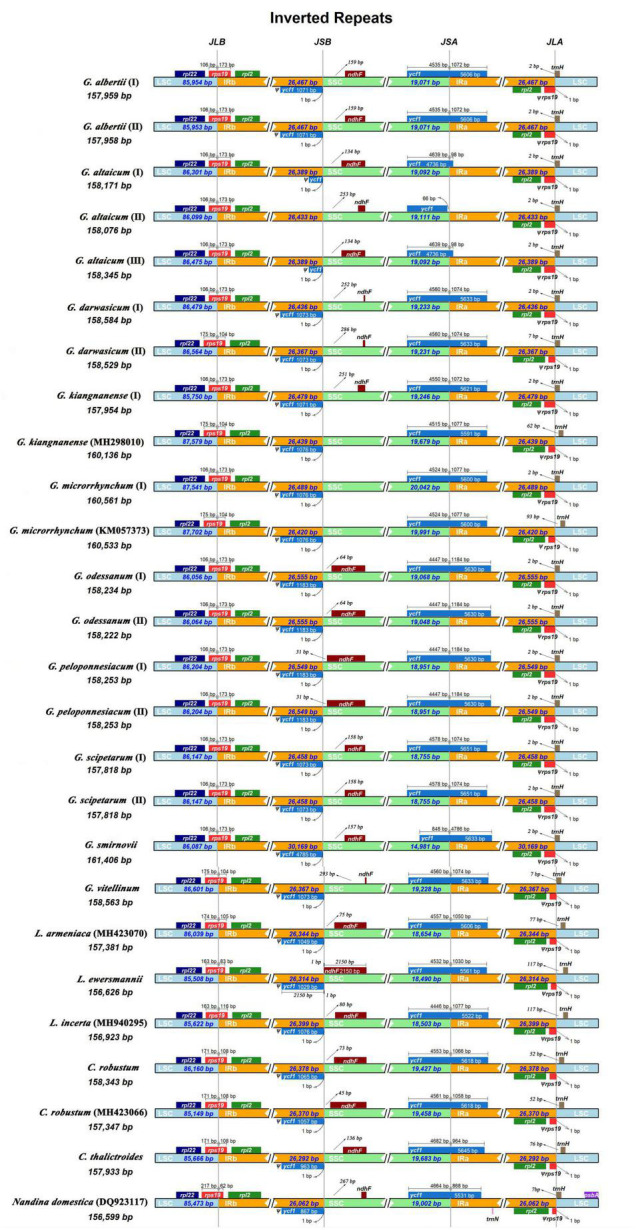
Comparison of the LSC/IRb/SSC/IRa junctions among the 26 complete plastomes of Nandinoideae.

All 26 plastomes of Nandinoideae had their LSC/IRb junctions located within the *rps*19 gene, and thus contained a ψ*rps*19 (62–173 bp) in IRa (Figure 3). In almost all plastomes, the SSC/IRa boundary was located within the *ycf*1 gene, except for *G. altaicum* (II), in which the SSC/IRa boundary was situated in the spacer region *ycf*1-*trn*N*-*GUU (Figure 3). Therefore, there was no ψ*ycf*1 in *G. altaicum* (II), while for the remaining 25 plastomes, there was a ψ*ycf*1 with different length. The shortest ψ*ycf*1 (98 bp) was found in two (of the three) *G. altaicum* accessions (I and III), and the longest (4,786 bp) in *G. smirnovii*; in the other 23 plastomes, the length of this pseudogene varied from 868 to 1,184 bp (Figure 3). Hence, within Nandinoideae, the plastome of *G. smirnovii* (161,406 bp) was significantly longer than other plastomes, and this is mainly due to the increased length of ψ*ycf*1, which resulted in a significant expansion of the respective IR regions.

Of all the 114 unique genes identified across the 26 Nandinoideae plastomes, 26 differed in length, mostly less than 10 bp, albeit with some exceptions (see Supplementary Tables 1, 2). Specifically, in all three accessions of *G. altaicum* (I–III), the 5′ end of the *ycf*1 gene was *ca.* 1,000 bp shorter (I and III: 919 bp; II: 1,127 bp) than in the other plastomes, i.e., the *ycf*1 genes were seriously deleted at the 5′ end of the three plastomes of *G. altaicum*. In *G. microrrhynchum* (I), the *rop*C1 gene was *ca.* 750 bp shorter than the other 25 Nandinoideae plastomes due to an insertion of “A” in the locus of 1214 bp/1314, which resulted in its premature termination. For all the plastomes of *Leontice* and *Gymnospermium*, the *ndh*F gene had a 75-bp deletion (in the middle part) when compared with that of *Nandina* and *Caulophyllum*, the two early diverging genera of Nandinoideae (see “Comparative Plastome Analysis and Identification of diversity hotspot regions”). The GC content of the 26 plastomes ranged from 37.7% to 38.2%, whereby the highest values occurred in the IR regions (41.5–43.2%), followed by the LSC (36.1–36.6%) and SSC (30.7–32.6%) regions (Table 2).

### Comparative Plastome Analysis and Identification of Diversity Hotspot Regions

**FIGURE 4:**
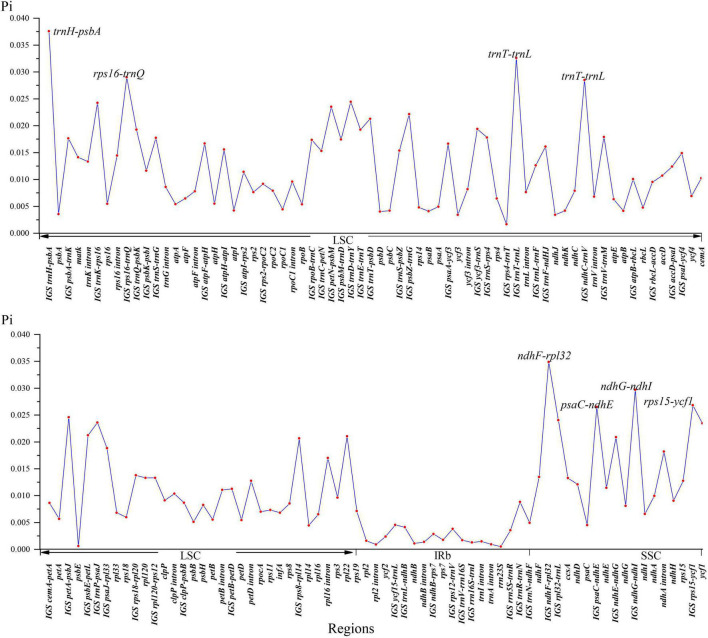
Nucleotide diversity (*Pi*) values of 26 Nandinoideae plastome sequences.

The global visualization alignment with mVISTA and MAUVE revealed that all the 26 Nandinoideae plastomes had a consistent gene order, whereby the IR regions showed a higher level of sequence identity than the two single-copy (SSC, LSC) regions (Supplementary Figures 1, 2). Across these 26 plastomes, we calculated levels of nucleotide diversity (*Pi* values) for a total of 137 regions, including 58 intergenic spacer (IGS) regions, 61 protein-coding (CDS) regions, 17 intron regions (of CDS/tRNA genes), and one *rRNA* gene (Figure 4). In general, the IGS regions exhibited higher levels of diversity than the CDS and intron regions. More specifically, for the 61 CDS regions, *Pi* ranged from 0.00061 (*psb*E) to 0.02346 (*ycf*1), yet only two genes, *ycf*1 and *rpl*22 showed remarkably high diversity (*Pi* > 0.02). By contrast, for the 58 IGS regions, *Pi* ranged from 0.00129 (*rrn*16S*-trn*I) to 0.03757 (*trn*H*-psb*A), and 19 of those showed remarkably high diversity (*Pi* > 0.02; i.e., *rps*8*-rp*l14, *ndh*E*-ndh*G, *psb*E*-pet*L, *trn*T*-psb*D, *psb*Z*-trn*G, *pet*N*-psb*M, *trn*P*-psa*J, *rpl*32*-trn*L, *trn*K*-rps*16, *trn*D*-trn*Y, *pet*A*-psb*J, *psa*C*-ndh*E, *rps*15*-ycf*1, *ndh*C*-trn*V, *rps*16*-trn*Q, *ndh*G*-ndh*I, *trn*T*-trn*L, *ndh*F*-rpl*32, and *trn*H*-psb*A; see Figure 4). The sizes and *Pi* values of these 21 hotspot regions were shown in Supplementary Table 3, and phylogenetic trees based on each region were shown in Supplementary Figure 3.

### Phylogenetic Analyses

**FIGURE 5:**
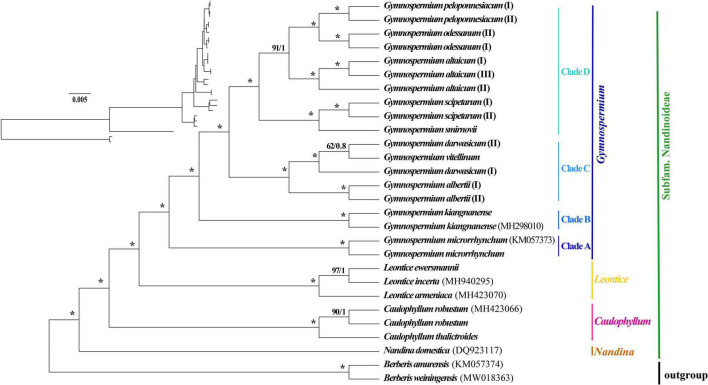
Phylogenetic tree reconstructions of 16 species of Nandinoideae (*n* = 26 accessions) plus two outgroup species of *Berberis* based on 80 plastome-derived CDS genes inferred from maximum likelihood (ML) and Bayesian inference (BI), respectively. Consecutive numbers at each node represent ML bootstrap support (BS) and BI posterior probability (PP) values, respectively. Asterisks indicate maximum support by both measures (i.e., BS = 100%/PP = 1).

The 80 plastome CDS regions of the 16 species (*n* = 26 accessions) of Nandinoideae and the two *Berberis* outgroup species were aligned with a total length of 70,192 bp. The topologies of the resulting ML and BI trees (Figure 5) were fully congruent and well resolved, with highly supported nodes. In fact, all three multi-species genera (*Caulophyllum*, *Leontice* and *Gymnospermium*) and all species with multiple accessions formed distinct clades, with both maximum ML bootstrap support (BS = 100%) and highest posterior probability (PP = 1). Only a few nodes (2 for ML tree vs. 4 for BI tree) in the tree received no full support. Within Nandinoideae, *Nandina* (*N. domestica*) was identified as the first diverging lineage, followed by *Caulophyllum* (represented by 2 spp./*n* = 3) and the sister genera *Leontice* (3 spp./*n* = 3) and *Gymnospermium* (10 spp./*n* = 19). Within *Leontice*, *L. armeniaca* was recovered as sister to *L. ewersmannii* + *L. incerta*. Within *Gymnospermium*, *G. microrrhynchum* (northeastern Asia) was the first diverging species (Clade A), followed by the East China endemic *G. kiangnanense* (Clade B). All the remaining species formed two highly supported sister clades (C, D), comprising three species from West Tianshan (Clade C: *G. albertii* as sister to *G. vitellinum* + *G. darwasicum*) vs. five species (Clade D) distributed in the Altai Mountains (*G. altaicum*), Caucasus Mountains (*G. smirnovii*), and southeastern Europe (*G. odessanum*, *G. peloponnesiacum*, *G. scipetarum*). Noticeably, within the subclade *G. darwasicum* (I and II) + *G. vitellinum*, the latter was embedded within the former, with low support (BS = 62%, PP = 0.8) at the node of *G. darwasicum* (II) and *G. vitellinum*. Within Clade D, *G. scipetarum* + *G. smirnovii* appeared to be a sister group to a subclade (BS = 91%, PP = 1) comprised of *G. altaicum* + *G. odessanum* + *G. peloponnesiacum*.

## Discussion

### Comparative Plastome Genomics

All the 20 plastomes of Nandinoideae newly sequenced in this study contained 114 unique genes and in the same order (Supplementary Table 1 and Supplementary Figures 1, 2). This is consistent with six previously reported plastomes of this subfamily (Moore et al., 2006; Sun et al., 2018; Yang et al., 2018; He et al., 2019), and also agrees with the gene content and order of plastomes of Podophylloideae (Ye et al., 2018). However, these Nandinoideae plastomes have one more gene (*rpo*A) than the genera of Berberidoideae, such as *Alloberberis* C. C. Yu and K. F. Chung, *Berberis*, *Mahonia*, and *Moranothamnus* C. C. Yu and K. F. Chung (Hsieh et al., 2022), and two more genes (*inf*A, *ycf*15) than *Epimedium* (Zhang et al., 2016), a member of Podophylloideae. In the present study, we also observed that the ψ*ycf*1 pseudogene of *Gymnospermium smirnovii* was ∼3,700 bp longer than in the 25 other Nandinoideae plastomes, which resulted in a significant IR expansion, as likewise found in several species of Berberidoideae and Podophylloideae (*Epimedium ecalcaratum* G. Y. Zhong, MN939634; *E. brevicornu* Maxim., MN371716) (Ma et al., 2013; Sun et al., 2018; Zheng et al., 2019; Hsieh et al., 2022). Insertions/deletions (indels) in the intergenic spacer (IGS) regions also contributed significantly to plastome length variations among species of Nandinoideae. As a case in point, the plastomes of two accessions of *Gymnospermium microrrhynchum* (I and KM057373) and one of *G. kiangnanense* (MH298010) were > 160,100 bp, while most other plastomes were < 158,600 bp, except for *G. smirnovii* (161,406 bp). Moreover, within *G. kiangnanense*, the plastome of accession ‘I’ was 2,180 bp shorter than that of ‘MH298010,’ mainly due to indels in the IGS regions. However, such large differences were not found in other species of Nandinoideae, and further study is needed to clarify the apparently dynamic evolution of the *G. kiangnanense* plastome. In general, indel and premature gene termination events are well known factors of plastome evolution in angiosperms (Fu et al., 2017; Wang et al., 2019; Zhang et al., 2020), including species of Berberidaceae (Sun et al., 2016, 2018; Hsieh et al., 2022). However, the severe truncation at the the 5′ end of the *ycf*1 gene in the three analyzed plastomes of *G. altaicum* plastomes (I and III: 919 bp; II: 1,127 bp), and the premature termination in the *rop*C1 gene have not been reported in the previous complete plastome analyses of Berberidaceae. Notably, a 75-bp deletion in the *ndhF* gene occurred in *Leontice* and *Gymnospermium*, which thus can be interpreted to present a molecular synapomorphy of these sister genera (Figure 5). As to another interesting observation, the length of the *rps*7 gene was consistently 468 bp across the 26 Nandinoideae plastomes analyzed, similar length (460 bp) with most genera of Berberidaceae (*Berberis*, *Mahonia*, *Achlys* DC., *Bongardia* C. A. Mey., *Vancouveria* C. Morren and Decne., *Epimedium* and *Jeffersonia* W. Bartram), while this gene is considerably short (63–78 bp) in some species of Podophylloideae (Zhang et al., 2016; Sun et al., 2018; Ye et al., 2018; Hsieh et al., 2022). On the other hand, significant variation in the length of the *acc*D gene has recently been reported for Berberidoideae (Hsieh et al., 2022), but this is not observed in Nandinoideae (Supplementary Table 2).

### Identification of Highly Variable Plastome Regions in Nandinoideae

Plastid markers combined with nuclear ribosomal DNA (ITS) sequences have proven useful in resolving the backbone phylogeny of Berberidaceae. Kim et al. (1996, 2004) identified the four clades of Beberidaceae consistent with the chromosomal groups (*n* = 7 for Berberidoideae, *n* = 6 for Podophylloideae, and *n* = 8/10 for Nandinoideae), as well as the systematic position of *Ranzania* T. Itô, *Bongardia*, *Diphylleia* Michx. and *Nandina* based on ITS, *rbc*L and *ndh*F. More recently, Yu and Chung (2017) proposed two new genera, *Alloberberis* and *Moranothamnus* (see “Comparative Plastome Genomics”), by concatenating ITS with plastid sequences of three coding regions (*acc*D, *ndh*F, *rbc*L) and one IGS region (*psb*A-*trn*H). Nevertheless, phylogenetic and phylogeographic studies within Nandinoideae are still rare. Barina et al. (2017) and Rosati et al. (2018) used ITS and different IGS plastid regions (*ndh*F-*trn*L and *trnL*-*trnF*, respectively) to explore the phylogenetic relationships of European *Gymnospermium* taxa, albeit with low levels of resolution. Our hotspot region analysis of whole plastomes disclosed that two coding genes (*rpl*22, *ycf*1) as well as 19 IGS regions (detailed in §3.2) had nucleotide diversity (*Pi*) values > 0.02. For seven of these 21 regions (*ycf 1*, *trnT-trnL*, *trnH-psbA*, *trnK-rps16*, *ndhF-rpl32*, *psbE-petL*, *rps15-ycf1*), the monophyly of all genera was successfully recovered (Supplementary Figure 3). Hence, these genes and regions, in particular, will be powerful molecular markers for future phylogenetic, phylogeographic and/or conservation genetic studies in Nandinoideae. Moreover, these markers should serve as ideal DNA barcodes to discriminate among the often rare, endangered, and medicinally important species of this subfamily (see also Chang et al., 2004; Doroftei and Mierla, 2007; Karl and Strid, 2009; Mikatadze-Pantsulaia et al., 2010; Abidkulova et al., 2021).

### Phylogenetic, Taxonomic and Biogeographic Inferences

Our phylogenomic ML and BI analyses of Nandinoideae, based on 80 plastome-derived CDS genes from 16 (out of *ca.* 20) species and 26 accessions, plus two species of *Berberis* as outgroup (Figure 5), is the most comprehensive phylogenetic study of this subfamily by now, and provided a robust resolution for both generic and species relationships. Our results revealed a sister relationship between *Gymnospermium* and *Leontice*, which is consistent with previous studies (Kim and Jansen, 1996; Wang et al., 2007; Sun et al., 2018; Hsieh et al., 2022). However, this result differs from previous morphological-cladistic studies (Loconte and Estes, 1989; Nickol, 1995), which inferred *Leontice* as sister to either *Caulophyllum* or *Bongardia*. Within *Leontice*, we identified *L. armeniaca* from Western Asia as sister to a clade comprising two other species from Central Asia (*L. ewersmannii*, *L. incerta*). In recent studies, *Gymnospermium maloi* Kit Tan & Shuka has been reduced to a heterotypic synonym of *G. scipetarum*, based on morphological, karyological and molecular evidence (Tan et al., 2011; Barina et al., 2015, 2017). Hence, our sampling of *Gymnospermium* contained all species except one, *G. silvaticum* from western Tianshan. Rosati et al. (2018) sampled six *Gymnospermium* species for molecular analysis based on ITS and *trn*L-F sequences, and identified Italian (southern Apennine) populations of *Gymnospermium* representing the genus’ western range limit, as a subspecies of *G. scipetarum* (subsp. *eddae* Rosati, Farris, Fascetti and Selvi). In an earlier phylogenetic study, Barina et al. (2017) had sampled seven species of *Gymnospermium* to infer the genus’ ‘European’ evolutionary history, but lacked particular sequence information for *G. microrrhynchum* (ITS) and *G. kiangnanense* (*ndh*F*-trn*L), and neither included any West Tianshan species. Our phylogeny (Figure 5) is the first to identify *G. microrrhynchum* (Clade A) from northeastern Asia as the genus’ earliest diverging species, followed by *G. kiangnanense* (Clade B) from eastern China (representing the genus’ southern range limit). Three species from West Tianshan (*G. albertii*, *G. darwasicum*, *G. vitellinum*) formed a highly supported derived clade (‘C’), with *G. vitellinum* showing closer relationships to the two samples of *G. darwasicum* (Figure 5). Indeed, based on morphological characters, *G. vitellinum* has previously been inferred to be most closely related to *G. darwasicum* (Kral, 1981), yet due to lack of resolution, our phylogenomic data cannot exclude the possibility that these two species might be conspecific and/or hybridizing (etc.). As sister to the West Tianshan Clade C, we identified a highly supported group (Clade ‘D’) of mostly (but not exclusively) westerly distributed species (see also Tian and Mullaj, 2001a,b; Barina et al., 2007; 2015; 2017; Rosati et al., 2014), including *G. smirnovii* (Caucasus Mountains) + *G. scipetarum* (central Albania, southern Montenegro, Italy/southern Apennines) as likely sister group of a subclade (BS = 91, PP = 1) comprising *G. altaicum* (Central Asia; Abidkulova et al., 2021) as putative sister to *G. odessanum* (northern Black Sea area) + *G. peloponnesiacum* (southern Greece/Peloponnese). Clearly, these relationships have to be treated with some caution given that one species of *Gymnospermium* is missing in our taxon sampling, and further studies are required to clarify their biogeographic history, but which already now seems to point at an east-to-west expansion. Considering the apparently close relationship between *G. peloponnesiacum* and *G. altaicum*, it is noteworthy they retained several distinct morphological features even when cultivated together in the Göteborg Botanical Garden (Karl and Strid, 2009). Moreover, with regard to the putative sister pair *G. smirnovii*/*G. scipetarum* (I, II), it is worth to recall that the plastome of only the former species accession shows a significant expansion of the IR regions (caused by *ycf*1 length variation); yet again a wider sampling within both species would be needed to clarify whether the presence/absence of this IR expansion could serve as a species-diagnostic molecular marker. Altogether, we suggest that the five species of Clade D should be considered as independent species, rather than subspecies or varieties of *G. altaicum* (Stearn et al., 1993; Tian and Mullaj, 2001a; Phitos et al., 2003; Doroftei and Mierla, 2007).

Considering broader, yet preliminary biogeographic inferences from our phylogeny (Figure 5), it seems possible that *Gymnospermium* originated in northeast Asia and subsequently spread southward and westward, resulting in the genus’ high species diversity seen today. Notably, in our field surveys in China (Pan Li and Zhaoping Yang, personal observation), species of the Eurasian/North American genus *Erythronium* L. (Liliaceae; *ca.* 27–32 spp.) were often found to be associated with *Gymnospermium*, for instance, *G. microrrhynchum* with *E. japonicum* Decne. in northeast China, and *G. altaicum* with *E. sibiricum* (Fisch. and C. A. Mey.) Krylov in the Altai Mountains of northwest China. Based on an earlier molecular (plastid/nuclear) phylogeny of *Erythronium* (Allen et al., 2003), there is relatively little divergence among the four Eurasian species (distributed from Portugal to Japan). In addition, we found many other spring ephemerals accompanying *Gymnospermium* and *Erythronium* in northeast and northwest China, such as *Gagea* Salisb. and *Tussilago* L., all of which share similar distribution patterns across Eurasia (Peterson et al., 2008; Chen and Nordenstam, 2011). Hence, it would be extremely interesting to clarify in future comparative phylogeographic studies whether all these spring ephemerals share a similar biogeographical history of east-to-west expansion routes across Eurasia, including joint areas of (glacial) refugial survival.

Finally, it worth to briefly review previous time estimates of divergence within Nandinoideae. For example, in their plastid (*rbc*L) study on plant (sister-) species pairs with disjunct distributions in East Asia (EA) and eastern North America (ENA), Xiang et al. (2000) estimated the divergence time between *Caulophyllum robustum* (EA) and *C. thalictroides* (ENA) to the Early Pleistocene, ca. 2.38 ± 1.69 million years ago (Mya). By contrast, Barina et al. (2017), using nuclear (ITS) and plastid sequences (*ndh*F-*trn*L), dated the split between *Leontice* and *Gymnospermium* (likely in East Asia) to the mid-Miocene, *ca.* 16.4 (32.7–7.4) Mya, while the onset of diversification the latter genus, including the westward spread of the *G. altaicum* lineage to the Black Sea area, likely occurred in the early Late Miocene, *ca.* 11.4 (23.4–4.8) Mya. Based on a plastid phylogenomic approach, Sun et al. (2018) dated the divergence of *Nandina* and the other three genera of *Nandinoideae* at 33–13 Mya, and *ca.* 5 Mya for *Leontice* and *Gymnospermium*. Altogether, these authors included only one species for each genus of Nandinoideae, and failed to reconstruct the ancestral areas of *Caulophyllum*, *Leontice*, and *Gymnospermium*, with uncertain ancestral area (Sun et al., 2018). As a result, the evolutionary history of Nandinoideae was still unclear. In total, these inferences of divergence times and dispersal routes were mainly based on a limited sampling of taxa and mostly plastid (Xiang et al., 2000; Sun et al., 2018), or more rarely, plastid and nuclear (ITS) data (Barina et al., 2017). Hence, further phylogenomic analyses of both plastome and nuclear genomic (or transcriptomic) data are required to fully uncover the evolutionary and biogeographic history of Nandinoideae.

## Data Availability Statement

The datasets presented in this study can be found in online repositories. The names of the repository/repositories and accession number(s) can be found in the article/Supplementary Material.

## Author Contributions

PL and ZY designed the research and provided the research resources. PL, DZ, ZY, SS, XL, XZ, and JL collected the plant materials. SS, HL, and ZY assembled and analyzed the data and prepared the figures and tables. ZY, SS, PL, and HC wrote the manuscript. All authors revised the manuscript.

## Conflict of Interest

The authors declare that the research was conducted in the absence of any commercial or financial relationships that could be construed as a potential conflict of interest.

## Publisher’s Note

All claims expressed in this article are solely those of the authors and do not necessarily represent those of their affiliated organizations, or those of the publisher, the editors and the reviewers. Any product that may be evaluated in this article, or claim that may be made by its manufacturer, is not guaranteed or endorsed by the publisher.

## Footnotes

1
http://www.geneious.com

2
https://www.phylo.org

## Supplementary Material

Supplementary Figure 1 Comparison of the 26 plastomes of Nandinoideae analyzed in this study using mVISTA, with *Ranzania japonica* (MG234280) as a reference.

Supplementary Figure 2 MAUVE alignment of the 26 Nandinoideae plastomes analyzed in this study, with *Gymnospermium albertii* (I) as a reference.

Supplementary Figure 3 Maximum likelihood (ML) trees based on each of the 21 hotspot regions (*Pi* > 0.02). **(A)** *ycf*1; **(B)** *trn*T*-trn*L; **(C)** *trn*T*-psb*D; **(D)** *rpl*32*-trn*L; **(E)** *trn*H*-psb*A; **(F)** *pet*N*-psb*M; **(G)** *trn*K*-rps*16; **(H)** *ndh*G*-ndh*I; **(I)** *ndh*F*-rpl*32; **(J)** *psb*E*-pet*L; **(K)** *ndh*C*-trn*V; **(L)** *rpl*22; **(M)** *pet*A*-psb*J; **(N)** *trn*P*-psa*J; **(O)** *rps*15*-ycf*1; **(P)** *rps*16*-trn*Q; **(Q)** *psb*Z*-trn*G; **(R)** *trn*D*-trn*Y; **(S)** *rps*8*-rpl*14; **(T)** *psa*C*-ndh*E; U, *ndh*E*-ndh*G. Numbers listed at each node represents bootstrap support (BS) values. The hyphen indicates BS < 50%.

## References

[B1] Abidkulova D. M. Ivashchenko A. A. Sramko G. Kurbatova N. V. Abidkulova K. T. (2021). *Gymnospermium altaicum* (Pall.) Spach (*Berberidaceae*), an early spring element of wild fruit forests of the Trans-Ili Alatau. *Exp. Biol.* 86 14–26. 10.26577/eb.2021.v86.i1.02

[B2] Allen G. A. Soltis D. E. Soltis P. S. (2003). Phylogeny and Biogeography of *Erythronium* (Liliaceae) inferred from chloroplast matK and nuclear rDNA ITS sequences. *Syst. Bot.* 28 512–523.

[B3] Al-Snafi A. E. (2019). Constituents and pharmacological effects of *Leontice leontopetalum* – a review. *Chem. J.* 3 103–108.

[B4] Amiryousefi A. Hyvonen J. Poczai P. (2018). IRscope: an online program to visualize the junction sites of chloroplast genomes. *Bioinformatics* 34 3030–3031. 10.1093/bioinformatics/bty220 29659705

[B5] Barina Z. Caković D. Pifkó D. Schönswetter P. Somogyi G. Frajman B. (2017). Phylogenetic relationships, biogeography and taxonomic revision of European taxa of *Gymnospermium* (*Berberidaceae*). *Bot. J. Linn. Soc.* 184 298–311. 10.1093/botlinnean/box028

[B6] Barina Z. Pifco D. Kiraly G. (2007) “Data on the flora of Griba Mountains (South-Albania),” in *Abstracts, 4th Balkan Botanical Congress, Sofia, 20–26 June 2006* (Sofia: Bulgarian Academy of Sciences), 265.

[B7] Barina Z. Pintér B. Pifkó D. (2015). Morphometrical studies on *Gymnospermium scipetarum* and G. maloi (*Berberidaceae*). *Wulfenia* 22 209–220.

[B8] Baytop T. (1984). *Therapy with Medicinal Plants in Turkey (Past and Present).* Istanbul: Istanbul University Press.

[B9] Chang C. S. Kim H. Park T. Y. Maunder M. (2004). Low levels of genetic variation among southern peripheral populations of the threatened herb. *Leontice microrhyncha* (*Berberidaceae*) in Korea. *Biol. Conserv.* 119 387–396. 10.1016/j.biocon.2003.12.003

[B10] Chen X. H. Xiang K. L. Lian L. Peng H. W. Erst A. S. Xiang X. G. (2020). Biogeographic diversification of *Mahonia* (*Berberidaceae*): implications for the origin and evolution of East Asian subtropical evergreen broadleaved forests. *Mol. Phylogenetics Evol.* 151:106910. 10.1016/j.ympev.2020.106910 32702526

[B11] Chen Y. L. Nordenstam B. (2011). “Asteraceae-Tussilago L,” in *Flora of China*, eds Wu Z. Y. Raven P. H. Hong D. Y. (St. Louis: Missouri Botanical Garden Press), 714–800.

[B12] Darling A. C. Mau B. Blattner F. R. Perna N. T. (2004). Mauve: multiple alignment of conserved genomic sequence with rearrangements. *Genome Res.* 14 1394–1403. 10.1101/gr.2289704 15231754 PMC442156

[B13] Darriba D. Taboada G. L. Doallo R. Posada D. (2012). jModelTest 2: more models, new heuristics and parallel computing. *Nat. Methods* 9:772. 10.1038/nmeth.2109 22847109 PMC4594756

[B14] Dierckxsens N. Mardulyn P. Smits G. (2017). NOVOPlasty: de novo assembly of organelle genomes from whole genome data. *Nucleic Acids Res.* 45:e18. 10.1093/nar/gkw955 28204566 PMC5389512

[B15] Doroftei M. Mierla M. (2007). *Gymnospermium altaicum* in northern Dobrogea. *Brukenthal Acta Musei* 2 49–54.

[B16] Duke J. A. (2002). *Handbook of Medicinal Herbs* (2*^nd^* Edition). New York, NY: CRC Press.

[B17] Erichsen-Brown C. (1989). *Medicinal and Other uses of North American Plants: a Historical Survey with Special Reference to the Eastern Indian Tribes.* New York, NY: Dover publications.

[B18] Frazer K. A. Pachter L. Poliakov A. Rubin E. M. Dubchak I. (2004). VISTA: computational tools for comparative genomics. *Nucleic Acids Res.* 32 W273–W279. 10.1093/nar/gkh458 15215394 PMC441596

[B19] Fu C. N. Li T. H. Milne R. Zhang T. Ma P. F. Yang J. (2017). Comparative analyses of plastid genomes from fourteen cornales species: inferences for phylogenetic relationships and genome evolution. *BMC Genom.* 18:956. 10.1186/s12864-017-4319-9 29216844 PMC5721659

[B20] Fukuda I. (1967). The biosystematics of *Achlys*. *Taxon* 16 308–316. 10.2307/1216381

[B21] Govaerts R. Nic Lughadha E. Black N. Turner R. Paton A. (2021). The world checklist of vascular plants, a continuously updated resource for exploring global plant diversity. *Sci. Data* 8:215 10.1038/s41597-021-00997-6 34389730 PMC8363670

[B22] Greiner S. Lehwark P. Bock R. (2019). OrganellarGenomeDRAW (OGDRAW) version 1.3.1: expanded toolkit for the graphical visualization of organellar genomes. *Nucleic Acids Res.* 47 W59–W64. 10.1093/nar/gkz238 30949694 PMC6602502

[B23] Gresser G. Bachmann P. Witte L. Czygan F. C. (1993). Distribution and taxonomic significance of quinolizidine alkaloids in *Leontice leontopetalum* and L. ewersmannii (*Berberidaceae*). *Biochem. Syst. Ecol.* 21 679–685. 10.1016/0305-1978(93)90072-Y

[B24] He P. Z. Ma Q. Dong M. F. Yang Z. P. Liu L. X. (2019). The complete chloroplast genome of *Leontice incerta* and phylogeny of *Berberidaceae*. *Mitochondrial DNA Part B* 4 101–102.

[B25] Hess H. E. Landolt E. Hirzel R. (1977). *Berberidaceae” in Flora der Schweiz und Angrenzender Gebiete.* Zurich: Birkhäuser Basel Press, 103–105.

[B26] Hsieh C. L. Yu C. C. Huang Y. L. Chung K. F. (2022). *Mahonia* vs. Berberis unloaded: generic delimitation and infrafamilial classification of *Berberidaceae* based on plastid phylogenomics. *Front. Plant Sci.* 12:720171. 10.3389/fpls.2021.720171 35069611 PMC8770955

[B27] Hutchinson J. (1920). Jeffersonia and Plagiorhegma. *Bull. Misc. Inform.* 1920 242–245. 10.2307/4107483

[B28] Ikuta A. Itokawa H. (1988). Alkaloids of tissue cultures of Nandina domestica. *Phytochemistry* 27 2143–2145.

[B29] Iskandarov S. Nuriddinov R. N. Yunusov S. Y. (1967). Alkaloids of *Leontice albertii*. *Chem. Nat. Compd.* 3 297–298. 10.1007/BF00574645

[B30] Karl R. Strid A. (2009). *Bongardia chrysogonum* (*Berberidaceae*) rediscovered on the East Aegean island of Chios. *Phytol. Balc.* 15 337–342.

[B31] Katoh K. Standley D. M. (2013). MAFFT multiple sequence alignment software version 7: improvements in performance and usability. *Mol. Biol. Evol.* 30 772–780. 10.1093/molbev/mst010 23329690 PMC3603318

[B32] Kim Y. D. Jansen R. K. (1996). Phylogenetic implications of rbcL and ITS sequence variation in the *Berberidaceae*. *Syst. Bot.* 21 381–396. 10.2307/2419666

[B33] Kim Y. D. Kim S. H. Kim C. H. Jansen R. K. (2004). Phylogeny of *Berberidaceae* based on sequences of the chloroplast gene ndhF. *Biochem. Syst. Ecol.* 32 291–301. 10.1016/j.bse.2003.08.002

[B34] Kolak U. Hacıbekiroðlu I. Boða M. Özgökçe F. Ünal M. Choudhary M. I. (2011). Phytochemical investigation of *Leontice leontopetalum* L. subsp. ewersmannii with antioxidant and anticholinesterase activities. *Rec. Nat. Prod.* 5 309–313.

[B35] Kral M. (1981). Gymnospermium vitellinum, a new species of the genus *Gymnospermium*. *Preslia* 53 67–68.

[B36] Liao M. C. Wang Y. W. Xiao P. G. (2001). Study on dioctive compounds of Leontice kiangnanensis. *Chin. J. Plant Sci.* 19 513–516.

[B37] Loconte H. Blackwell W. H. (1985). Intrageneric taxonomy of Caulophyllum (*Berberidaceae*). *Rhodora* 87 463–469.

[B38] Loconte H. Estes J. R. (1989). Generic relationships within Leonticeae (*Berberidaceae*). *Can. J. Bot.* 67 2310–2316. 10.1139/b89-295

[B39] Ma J. Yang B. Zhu W. Sun L. Tian J. Wang X. (2013). The complete chloroplast genome sequence of *Mahonia bealei* (*Berberidaceae*) reveals a significant expansion of the inverted repeat and phylogenetic relationship with other angiosperms. *Gene* 528 120–131. 10.1016/j.gene.2013.07.037 23900198

[B40] Mikatadze-Pantsulaia T. Barblishvili T. Trivedi C. Kikodze D. Khutsishvili M. (2010). Ex situ conservation of some endemic and protected plant species in Georgia. *Kew Bull.* 65 643–648.

[B41] Moore M. J. Dhingra A. Soltis P. S. Shaw R. Farmerie W. G. Folta K. M. (2006). Rapid and accurate pyrosequencing of angiosperm plastid genomes. *BMC Plant Biol.* 6:17. 10.1186/1471-2229-6-17 16934154 PMC1564139

[B42] Nickol M. G. (1995). Phylogeny and inflorescences of *Berberidaceae* –a morphological survey. *Plant Syst. Evol.* 9 327–340. 10.1007/978-3-7091-6612-3_35

[B43] Peng C. Y. Liu J. Q. Zhang R. Shu J. C. (2014). A new alkaloid from the fruit of *Nandina domestica* Thunb. *Nat. Prod. Res.* 28 1159–1164. 10.1080/14786419.2014.921166 24897106

[B44] Peng Y. (2006). A pharmacophylogenetic study of the *Berberidaceae* (s.l.). *Acta Phytotax. Sin.* 44 241–257. 10.1360/aps040149

[B45] Peterson A. Levichev I. G. Peterson J. (2008). Systematics of *Gagea* and Lloydia (*Liliaceae*) and infrageneric classification of gagea based on molecular and morphological data. *Mol. Phylogenet. Evol.* 46 446–465. 10.1016/j.ympev.2007.11.016 18180173

[B46] Phitos D. Strid A. Tan K. (2003). “*Gymnospermium* Spach,” in *Flora Hellenica*, eds Strid A. Tan K. (Hessen: Koeltz Botanical Books Press), 81–82.

[B47] Rader J. I. Pawar R. S. (2013). Primary constituents of blue cohosh: quantification in dietary supplements and potential for toxicity. *Anal. Bioanal. Chem.* 405 4409–4417. 10.1007/s00216-013-6783-7 23420136

[B48] Ronquist F. Teslenko M. Van Der Mark P. Ayres D. L. Darling A. Hohna S. (2012). MrBayes 3.2: efficient Bayesian phylogenetic inference and model choice across a large model space. *Syst. Biol.* 61 539–542. 10.1093/sysbio/sys029 22357727 PMC3329765

[B49] Rosati L. Coppi A. Farris E. Fascetti S. Becca G. Peregrym M. (2018). The genus *Gymnospermium* (*Berberidaceae*) in Italy: identity and relationships of the populations at the western limit of the genus range. *Plant Biosyst.* 153 796–808. 10.1080/11263504.2018.1549613

[B50] Rosati L. Farris E. Tilia A. Potenza G. Fascetti S. (2014). “*Gymnospermium scipetarum* (*Berberidaceae*) specie nuova per la flora italiana,” in *Floristica, Sistematica ed Evoluzione*, Comunicazioni, eds Peruzzi L. Domina G. (Rome: Societa Botanica Italiana Press), 7–8.

[B51] Rozas J. Ferrer-Mata A. Sanchez-Delbarrio J. C. Guirao-Rico S. Librado P. Ramos-Onsins S. E. (2017). DnaSP 6: DNA sequence polymorphism analysis of large data sets. *Mol. Biol. Evol.* 34 3299–3302. 10.1093/molbev/msx248 29029172

[B52] Shi L. Chen H. Jiang M. Wang L. Wu X. Huang L. (2019). CPGAVAS2, an integrated plastome sequence annotator and analyzer. *Nucleic Acids Res.* 47 W65–W73. 10.1093/nar/gkz345 31066451 PMC6602467

[B53] Son Y. An Y. Jung J. Shin S. Park I. Gwak J. (2019). Protopine isolated from *Nandina domestica* induces apoptosis and autophagy in colon cancer cells by stabilizing p53. *Phytother Res.* 33 1689–1696. 10.1002/ptr.6357 30932278

[B54] Stamatakis A. (2014). RAxML version 8: a tool for phylogenetic analysis and post-analysis of large phylogenies. *Bioinformatics* 30 1312–1313. 10.1093/bioinformatics/btu033 24451623 PMC3998144

[B55] Stearn W. T. (1938). Epimedium and Vancouveria (*Berberidaceae*), a monograph. *Bot. J. Linn. Soc.* 51 409–535. 10.1111/j.1095-8339.1937.tb01914.x

[B56] Stearn W. T. Webb D. A. Tutin T. G. Heywood V. H. Burges N. A. Valentine D. H. (1993). “*Gymnospermium* Spach,” in *Flora Europaea*, ed. Tutin T. G. (Cambridge: Cambridge University Press), 29–295.

[B57] Sun Y. Moore M. J. Landis J. B. Lin N. Chen L. Deng T. (2018). Plastome phylogenomics of the early-diverging eudicot family *Berberidaceae*. *Mol. Phylogenet. Evol.* 128 203–211. 10.1016/j.ympev.2018.07.021 30076981

[B58] Sun Y. Moore M. J. Zhang S. Soltis P. S. Soltis D. E. Zhao T. (2016). Phylogenomic and structural analyses of 18 complete plastomes across nearly all families of early-diverging eudicots, including an angiosperm-wide analysis of IR gene content evolution. *Mol. Phylogenet. Evol.* 96 93–101. 10.1016/j.ympev.2015.12.006 26724406

[B59] Tabatadze N. Bun S. S. Tabidze B. Mshvildadze V. Dekanosidze G. Ollivier E. (2010). New triterpenoid saponins from *Leontice smirnovii*. *Fitoterapia* 81 897–901. 10.1016/j.fitote.2010.05.021 20554004

[B60] Tian K. Mullaj A. (2001a). “*Gymnospermium altaicum* subsp. *scipetarum*,” in *Med-Checklist Notulae*, eds Greuter W. Raus T. H. (Berlin: Willdenowia Press), 319–320.

[B61] Tian K. Mullaj A. (2001b). “Gymnospermium altaicum,” in *Endemic plants of Greece – The Peloponnese*, eds Tan K. Iatrou G. (Copenhagen: Gad Publishers), 138. 10.1186/2241-5793-21-9

[B62] Tan K. Shuka L. Siljak-Yakovlev S. Malo S. Pustahija F. (2011). The genus *Gymnospermium* (*Berberidaceae*) in the Balkans. *Phytotaxa* 25 1–17. 10.11646/PHYTOTAXA.25.1.1

[B63] Wang H. X. Liu H. Moore M. J. Landrein S. Wang H. F. (2019). Plastid phylogenomic insights into the evolution of the Caprifoliaceae *s.l.* (Dipsacales). *Mol. Phylogenet. Evol.* 142:106641. 10.1016/j.ympev.2019.106641 31605813

[B64] Wang W. Chen Z. D. Liu Y. Li R. Q. Li J. H. (2007). Phylogenetic and biogeographic diversification of *Berberidaceae* in the Northern Hemisphere. *Syst. Bot.* 32 731–742.

[B65] Xiang Q.-Y. Soltis D. E. Soltis P. S. Manchester S. R. Crawford D. J. (2000). Timing the eastern Asian–eastern North American floristic disjunction: molecular clock corroborates paleontological estimates. *Mol. Phylogenet. Evol.* 15 462–472. 10.1006/mpev.2000.0766 10860654

[B66] Yang Z. Peng Z. Zhang H. Lee J. Liu X. Fu C. (2018). The complete chloroplast genome of Gymnospermium kiangnanense (*Berberidaceae*): an endangered species endemic to Eastern China. *Mitochondrial DNA B: Resour.* 3 713–714. 10.1080/23802359.2018.1483760 33474293 PMC7799652

[B67] Ye W. Q. Yap Z. Y. Li P. Comes H. P. Qiu Y. X. (2018). Plastome organization, genome-based phylogeny and evolution of plastid genes in Podophylloideae (*Berberidaceae*). *Mol. Phylogenet. Evol.* 127 978–987. 10.1016/j.ympev.2018.07.001 29981470

[B68] Ying J. Boufford D. E. Brach A. R. (2011). “Berberidaceae,” in *Flora of China*, eds Wu Z. Y. Raven P. H. Hong D. Y. (St. Louis: Missouri Botanical Garden Press), 714–800.

[B69] Ying T. S. (1979). On Dysosma Woodson and Sinopodophyllum Ying, gen. nov. of the *Berberidaceae*. *Acta Phytotax. Sin.* 17 15–23.

[B70] Ying T. S. Terabayashi S. Boufford D. E. (1984). A monograph of Diphylleia (*Berberidaceae*). *J. Arnold Arbor.* 65 57–94.

[B71] Yu C. C. Chung K. F. (2017). Why *Mahonia*? Molecular recircumscription of *Berberis* s.l., with the description of two new genera, Alloberberis and Moranothamnus. *Taxon* 66 1371–1392. 10.12705/666.6

[B72] Zhang D. Gao F. Li W. X. Jakovlić I. Zou H. Zhang J. (2018). PhyloSuite: an integrated and scalable desktop platform for streamlined molecular sequence data management and evolutionary phylogenetics studies. *Mol. Ecol. Resour.* 20 348–355. 10.1111/1755-0998.13096 31599058

[B73] Zhang M. L. Uhink C. H. Kadereit J. W. (2007). Phylogeny and biogeography of *Epimedium*/ *Vancouveria* (*berberidaceae*): Western North american - East Esian disjunctions, the origin of european mountain plant taxa, and east asian species diversity. *Syst. Bot.* 32 81–92. 10.1600/036364407780360265

[B74] Zhang R. Wang Y. H. Jin J. J. Stull G. W. Anne B. Domingos C. (2020). Exploration of plastid phylogenomic conflict yields new insights into the deep relationships of leguminosae. *Syst. Biol.* 69, 613–622. 10.1093/sysbio/syaa013 32065640 PMC7302050

[B75] Zhang Y. Du L. Liu A. Chen J. Wu L. Hu W. (2016). The complete chloroplast genome sequences of five *Epimedium* species: lights into phylogenetic and taxonomic analyses. *Front. Plant Sci.* 7:306. 10.3389/fpls.2016.00306 27014326 PMC4791396

[B76] Zheng G. Zhang C. Yang J. Xu X. (2019). Characterization of the complete chloroplast genome of (*Berberidaceae*). *Mitochondrial DNA B: Resour.* 4 3681–3682. 10.1080/23802359.2019.1678429 33366141 PMC7707583

[B77] Zunnunzhanov A. Iskandarov S. Yunusov S. Y. (1971). Darvasamine – a new alkaloid from *Leontice darvasica*. *Chem. Nat. Compd.* 7 838–839. 10.1007/BF00567973

