# Supplementary figures and images for: Plastid Phylogenomics and Plastome Evolution of Nandinoideae (Berberidaceae)
Source: Front Plant Sci. 2022 Jun 30;13:913011. doi: 10.3389/fpls.2022.913011 (PMC9302238; doi:10.3389/fpls.2022.913011)

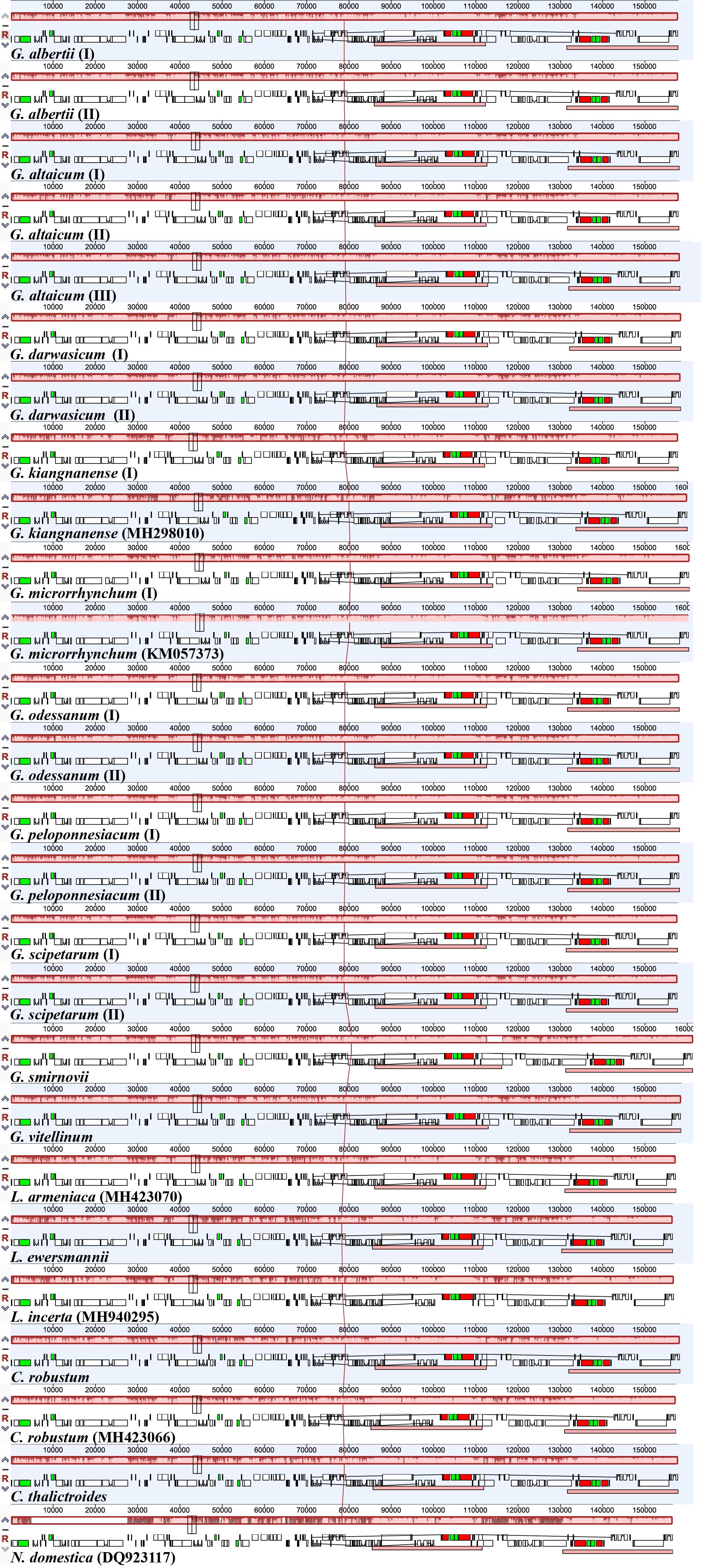

Supplement: Supplementary Figure 1 — Comparison of the 26 plastomes of Nandinoideae analyzed in this study using mVISTA, with Ranzania japonica (MG234280) as a reference. [file Image_1.JPEG]

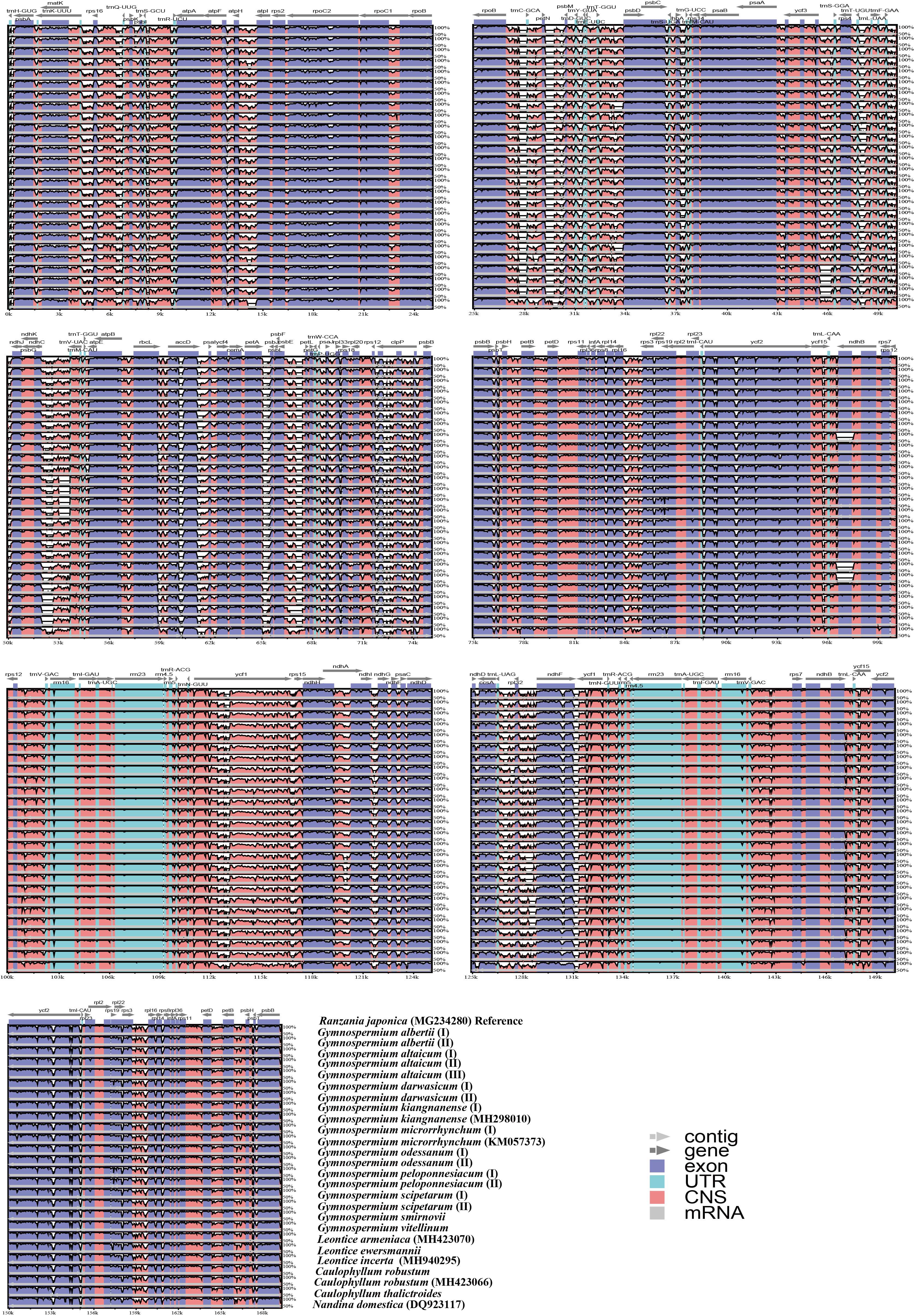

Supplement: Supplementary Figure 2 — MAUVE alignment of the 26 Nandinoideae plastomes analyzed in this study, with Gymnospermium albertii (I) as a reference. [file Image_2.JPEG]
