# Supplementary material for: Plastid Phylogenomics and Plastome Evolution of Nandinoideae (Berberidaceae)
Source: Front Plant Sci. 2022 Jun 30;13:913011. doi: 10.3389/fpls.2022.913011 (PMC9302238; doi:10.3389/fpls.2022.913011)
Supplement: Supplementary file 6 [file Table_3.DOCX]

**Table S3.** Sizes and *Pi* values of 21 hotspot regions (*Pi* > 0.02).

| Regions | Number of sites | Eta (Number of mutations) | *Pi* |
| --- | --- | --- | --- |
| CDS *ycf1* | 4807 | 683 | 0.02346 |
| IGS *trnT-trnL* | 1495 | 78 | 0.03258 |
| IGS *trnT-psbD* | 1329 | 136 | 0.02129 |
| IGS *rpl32-trnL* | 1220 | 96 | 0.02405 |
| IGS *trnH-psbA* | 1154 | 80 | 0.03757 |
| IGS *petN-psbM* | 1142 | 142 | 0.02350 |
| IGS *trnK-rps16* | 828 | 111 | 0.02424 |
| IGS *ndhG-ndhI* | 712 | 63 | 0.02971 |
| IGS *ndhF-rpl32* | 668 | 83 | 0.03490 |
| IGS *psbE-petL* | 649 | 86 | 0.02122 |
| IGS *ndhC-trnV* | 560 | 91 | 0.02849 |
| CDS *rpl22* | 534 | 86 | 0.02104 |
| IGS *petA-psbJ* | 525 | 69 | 0.02459 |
| IGS *trnP-psaJ* | 466 | 52 | 0.02360 |
| IGS *rps15-ycf1* | 456 | 60 | 0.02682 |
| IGS *rps16-trnQ* | 442 | 71 | 0.02902 |
| IGS *psbZ-trnG* | 404 | 34 | 0.02213 |
| IGS *trnD-trnY* | 316 | 60 | 0.02443 |
| IGS *rps8-rpl14* | 295 | 22 | 0.02067 |
| IGS *psaC-ndhE* | 295 | 51 | 0.02651 |
| IGS *ndhE-ndhG* | 268 | 30 | 0.02087 |
